# Esophageal Perforation Following Small-Bore Nasogastric Tube Placement: A Case Report

**DOI:** 10.7759/cureus.106485

**Published:** 2026-04-05

**Authors:** Kate Sanner Dixon, Alexis Ortega, Sofie Hass, Erich Wessel

**Affiliations:** 1 Surgery, University of Kansas Medical Center, Kansas City, USA; 2 Surgery, Kansas City University of Medicine and Biosciences, Kansas City, USA

**Keywords:** enteral feeding complications, esophageal perforation, gastric perforation, iatrogenic injury, nasogastric tube placement

## Abstract

Esophageal perforations are associated with high morbidity and mortality and can have iatrogenic or spontaneous causes. Nasogastric tube intubation, while generally considered a safe procedure, can be a very rare iatrogenic cause. A 61-year-old male underwent direct laryngoscopy, total laryngectomy, and esophageal dilation with complex closure for laryngeal obstruction and tracheocutaneous fistula secondary to laryngeal cancer. He experienced an esophageal perforation secondary to nasogastric tube advancement and required emergent surgical exploration via thoracotomy and laparotomy with washout and gastrostomy tube placement. Notably, the tube penetrated the cervical esophageal mucosa, tracked distally within a submucosal plane, and ultimately perforated the stomach at the gastroesophageal junction. He subsequently experienced recurrence of his esophageal strictures and became gastrostomy tube dependent. Signs and symptoms of esophageal perforations can present in a vague and nonspecific manner, mimicking those of other disorders. When a perforation is suspected, a chest X-ray can be used to identify early clues, and a contrast esophagogram and CT scan can be used to confirm the diagnosis. Although uncommon, esophageal perforation should be considered in any patient who develops acute symptoms following nasogastric tube manipulation. Prompt diagnosis and early surgical intervention are essential for optimal outcomes.

## Introduction

Esophageal perforation is a life-threatening condition associated with high morbidity and mortality [1]. The causes can be iatrogenic or spontaneous and can occur in various parts of the esophagus, with the thoracic region being the most common, followed by the cervical region and then the abdominal region [2]. Seventy percent of esophageal perforations are iatrogenic and can be caused by instrumentation for diagnostic or therapeutic purposes, such as endoscopy, stent placement, hemostasis, foreign body removal, pneumatic dilation, and, rarely, nasogastric tube intubation [1,3,4].

Nasogastric tube intubation is performed to provide early enteral feeding, administer medication, or perform gastric decompression in patients who are otherwise unable to tolerate oral intake [1,2]. These tubes are often placed in intensive care units in critically ill patients, and insertion is traditionally performed blindly. Although placement is generally considered safe [5,2], nasogastric tube intubation can, in rare instances, lead to esophageal perforation, with approximately 3.1 cases per 1,000,000 reported annually [2,6]. Due to its rarity and nonspecific presentation, diagnosis and treatment are often delayed [3,4]. Risk factors for esophageal perforation include diabetic ketoacidosis (DKA), alcohol use, substance use, malnutrition, and critical illness. Esophageal candidiasis infection has also been shown to compromise esophageal mucosal integrity and predispose patients to perforation [6].

The reported mortality for treated esophageal perforations is 10-25% when therapy is initiated within 24 hours and 40-60% when treatment is delayed to or beyond 48 hours [4,6]. This increased mortality is attributed to the esophagus’s anatomic location, which allows rapid contamination of the mediastinum by bacteria and digestive enzymes, leading to the development of severe mediastinitis, empyema, sepsis, and multiple organ dysfunction [4]. Early clinical suspicion and prompt imaging are essential for timely management and prevention of severe complications [7]. This report describes a rare case of esophageal perforation following blind bedside advancement of a small-bore nasogastric feeding tube - Corpak (Avanos Medical, Inc., Georgia, USA) with submucosal esophageal tracking and distal gastric perforation, an injury pattern not previously described in the literature.

## Case presentation

**Figure 1:**
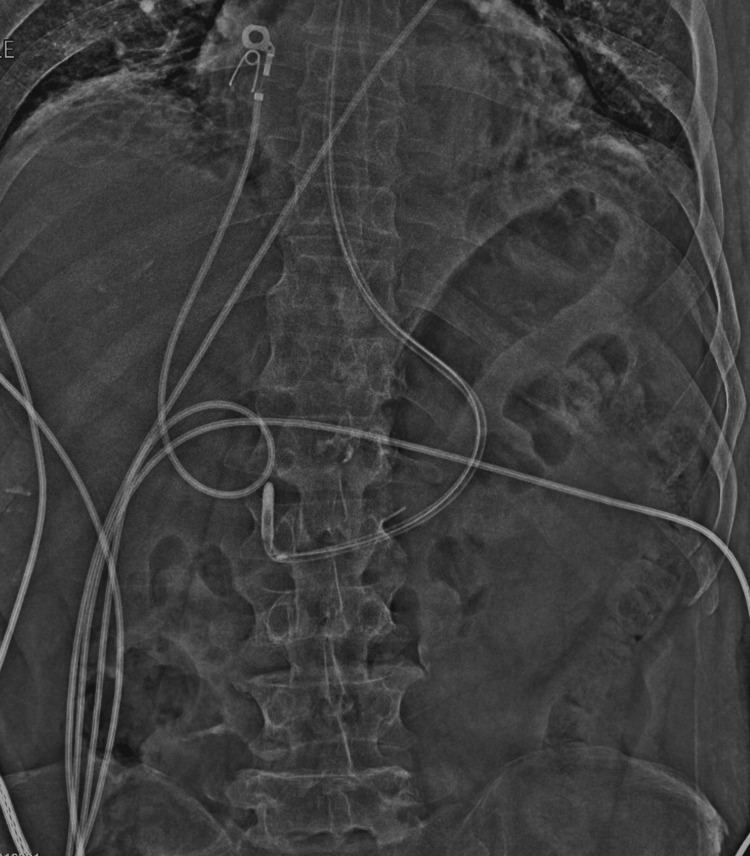
Abdominal radiograph confirming intragastric positioning of the small-bore nasogastric feeding tube (Corpak) with guidance stylet.

In March 2024, a 61-year-old male underwent direct laryngoscopy, total laryngectomy, and esophageal dilation with closure of the surgical defect for laryngeal obstruction and tracheocutaneous fistula secondary to chemoradiation for laryngeal cancer. He was noted to have esophageal stenosis requiring dilation in the setting of prior chemoradiation therapy. Given the nature of his procedure, he required postoperative enteral access bypassing the oral cavity to allow for appropriate healing. At the time of nasogastric tube advancement, there was no documented open proximal esophagostomy, and the injury involved the cervical esophageal mucosa. A small-bore Corpak nasogastric feeding tube was placed intraoperatively; however, it required further advancement postoperatively at the bedside in a blind fashion to achieve appropriate positioning. Placement was verified with plain film imaging (Figure 1), and small-volume tube feeds were initiated. The radiograph appeared to demonstrate appropriate distal positioning of the tube within the expected gastric silhouette. Shortly thereafter, the patient developed acute-onset abdominal pain and hemodynamic instability. 

**Figure 2:**
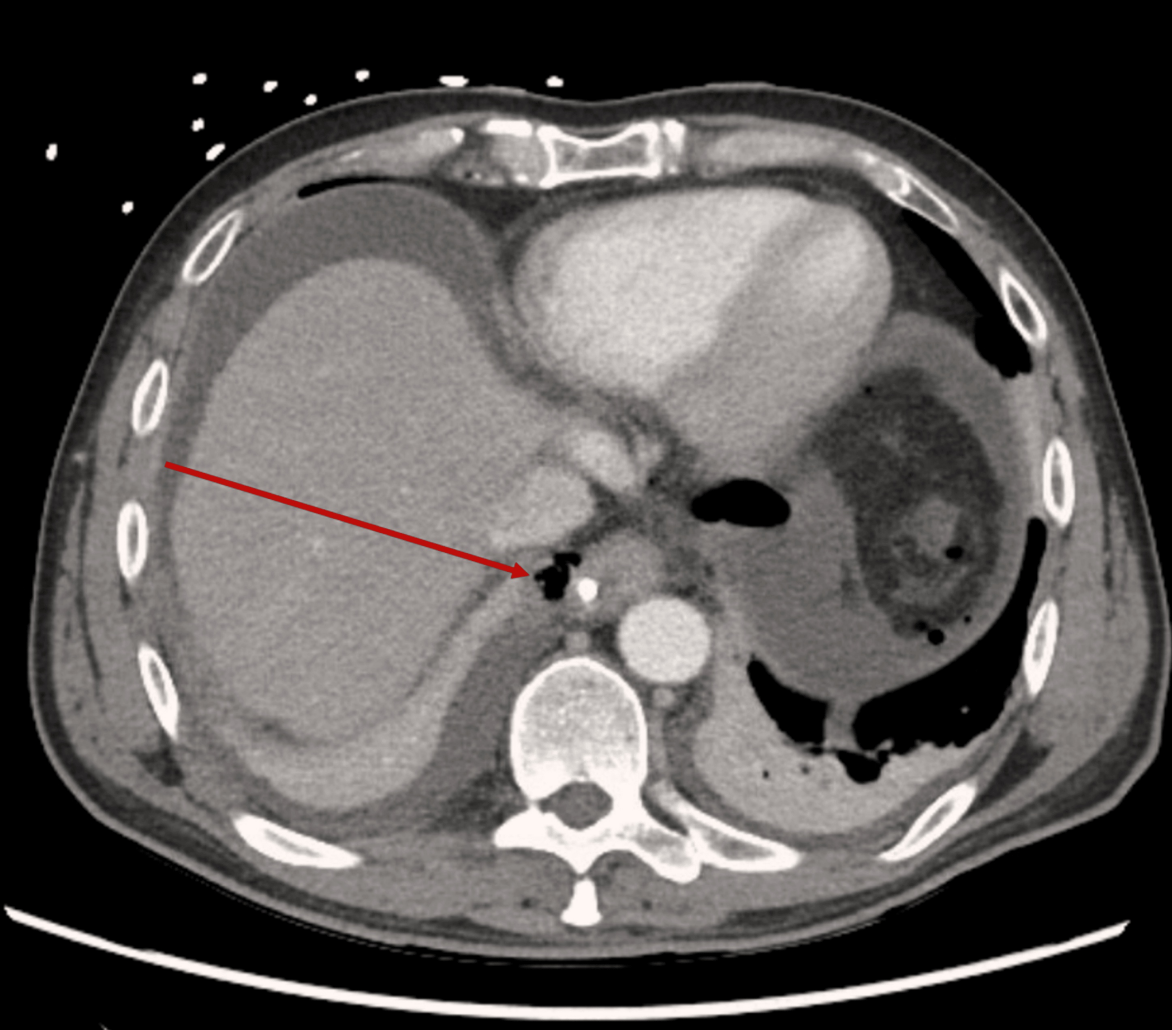
Axial computed tomography image demonstrating nasogastric tube perforating the distal esophagus with associated free air (red arrow).

**Figure 3:**
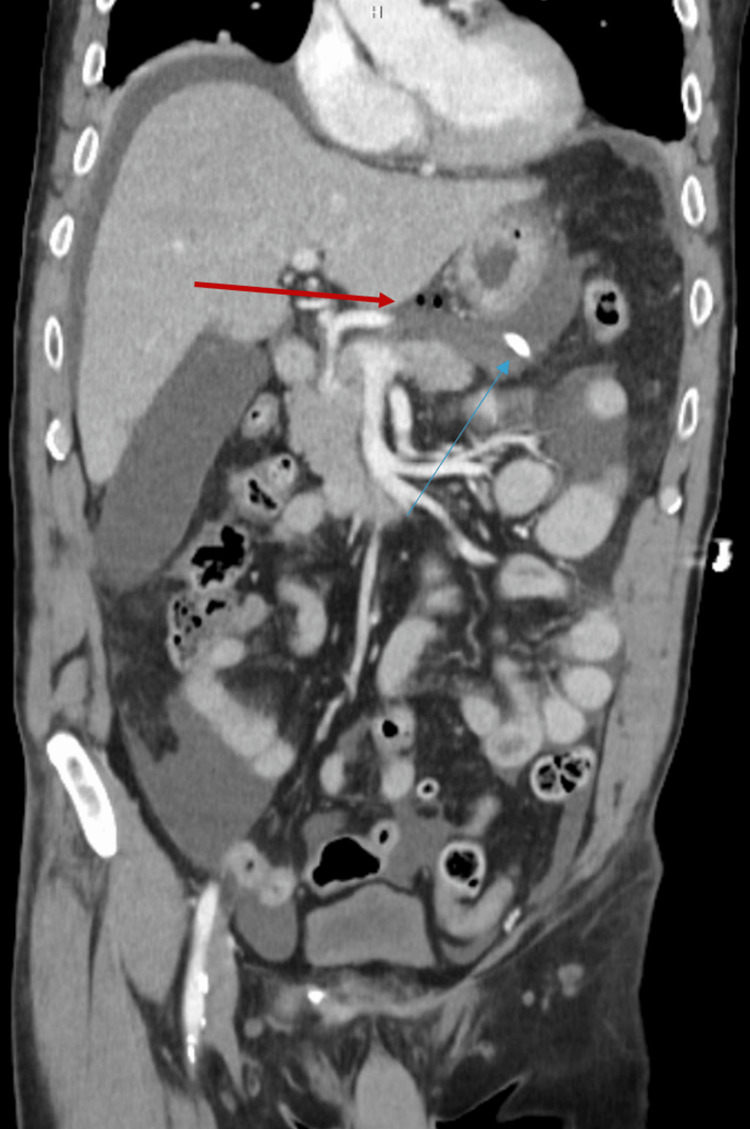
Coronal computed tomography image demonstrating the nasogastric tube extending from the distal esophagus across the gastroesophageal junction into the proximal gastric wall (blue arrow) with associated free air (red arrow).

A CT scan demonstrated the nasogastric tube traversing the distal esophagus with extension through the gastroesophageal junction and intraperitoneal positioning of the distal tip, with associated free air (Figures 2, 3). The patient was taken emergently to the operating room for surgical exploration. The esophagus was initially evaluated via left-sided thoracotomy and found to be injured distally, inferior to the diaphragm. The abdomen was then prepped and draped in sterile fashion, and an upper midline laparotomy was performed for intra-abdominal exploration as well as retrograde endoscopy via a controlled gastrotomy. Retrograde endoscopic evaluation via controlled gastrotomy demonstrated that the Corpak had penetrated the cervical esophageal mucosa and was coursing along a submucosal esophageal flap, ultimately exiting through the gastroesophageal junction and perforating the gastric wall with intraperitoneal extension. A multidisciplinary decision was made to keep the Corpak in place temporarily to facilitate identification of the injury for subsequent repair. The iatrogenic gastrotomy was primarily closed, and a gastrojejunostomy (GJ) tube was placed for enteral feeding. 

The patient required ICU-level care postoperatively, and the Corpak was removed at the bedside on postoperative day one after definitive operative management and stabilization. Tube feeds were gradually advanced via the GJ tube, with progression complicated by intermittent ileus with nausea, vomiting, and abdominal cramping. He was eventually discharged to long-term acute care and remained GJ tube-dependent due to esophageal stricturing, requiring serial dilations and supplemental tube feeds. Unfortunately, the patient passed away following a suicide attempt approximately one year after the case presentation.

## Discussion

Nasogastric feeding tube placement is a common bedside procedure often performed by nursing staff. Nasogastric tubes are flexible, hollow-bore devices passed through the nasal cavity into the stomach, duodenum, or jejunum and are used for enteral feeding, medication administration, gastric decompression, and stomach lavage [1,2,5]. They are frequently placed in critically ill patients with compromised oral intake to allow for adequate nutrition. The gold standard for confirming appropriate nasogastric tube position is an anteroposterior chest radiograph [5].

This case describes a rare esophageal injury with associated gastric perforation resulting from nasogastric tube intubation. Esophageal injury is an uncommon complication of nasogastric tube placement, and to our knowledge, there are no previously reported cases describing distal esophageal perforation with extension across the gastroesophageal junction into the proximal stomach resulting from submucosal esophageal tracking of a nasogastric tube [2,6]. Prior chemoradiation therapy and esophageal dilation may have contributed to mucosal fragility and altered tissue planes, predisposing the esophagus to submucosal dissection by a small-bore feeding tube. Similar complications have been reported with other small-bore feeding tubes, suggesting that reduced diameter alone does not eliminate the risk of iatrogenic injury in high-risk patients. Esophageal perforations carry significant morbidity and mortality, with a mortality rate of 10-25% when treated within 24 hours and 40-60% when treatment is delayed beyond 48 hours [4,6]. Approximately 70% of esophageal perforations are iatrogenic secondary to instrumentation, while spontaneous perforations, foreign bodies, and trauma account for 15%, 8%, and 5% of cases, respectively [4].

Clinical presentation of esophageal injury is often nonspecific and can mimic conditions such as pneumonia, angina, peptic ulcer disease, or pancreatitis, leading to diagnostic and therapeutic delays. Typical symptoms following esophageal instrumentation that would prompt injury concern include pain in the neck, chest, back, or epigastrium, as well as dysphagia, odynophagia, dysphonia, and dyspnea [8,9]. Common clinical signs include subcutaneous emphysema, shock, pneumothorax, hemothorax, fever, tachypnoea, tachycardia, and hypotension [4,10,11]. Physical exam findings may reveal crepitus on the chest, neck, or face, neck swelling, epigastric tenderness, or nasal speech, though findings may also be minimal or absent [8]. Presentation depends on timing from the injury to diagnosis and treatment, perforation location, degree of contamination, etiology, and associated mediastinal injury [1,7,10].

When esophageal perforation is suspected, a chest X-ray should be obtained, as it provides diagnostic clues in 70-90% of cases [7]. Early findings may include mediastinal emphysema, pleural effusion, or free intra-abdominal air [8]. A contrast esophagogram should be performed immediately if there is any suspicion of esophageal perforation [4]. This imaging modality is essential in all cases of suspected esophageal perforation, particularly those involving the middle or distal esophagus, as it confirms the diagnosis, localizes the site of perforation, evaluates communication with cervical and mediastinal spaces, and helps guide management decisions [7]. CT imaging has also proven effective in early diagnosis, particularly when classic signs are absent, as demonstrated in this case [12]. Given the diagnostic CT findings and the patient’s clinical instability, a contrast esophagogram was not performed. CT scans help identify perforation level and associated complications such as pneumothorax, pneumomediastinum, subcutaneous emphysema, or abscess formation [7].

Management for an esophageal perforation should be initiated immediately and includes intravenous fluids, nothing by mouth status, broad-spectrum antibiotics (for seven to 10 days), antifungals, proton pump inhibitors, analgesia, total parenteral nutrition, and evaluation for surgical versus non-operative management [3,4,6]. Surgical intervention is indicated in cases of clinical instability and is ideally performed within 24 hours, as the survival rate goes down when treatment is delayed [8,10,12]. Although primary esophageal repair was not required in this patient due to the injury pattern, full-thickness esophageal defects are typically repaired using a two-layer closure of mucosa and muscularis. All esophageal repairs should be drained by a large-bore intercostal chest tube, and a feeding jejunostomy should be added for nutrition [4]. Conservative non-operative management, such as making the patient nil per os (NPO), giving antibiotics, and close monitoring for self-resolution, may be successful in stable patients without signs of sepsis, including those with delayed or late-presenting lesions [1,12]. Emerging endoluminal procedures such as endoscopic vacuum therapy, endoscopic stent placement, endoscopic clip closure, endoscopic suturing, and tissue adhesives are becoming increasingly utilized for esophageal perforations, but were not appropriate in this case, given the extension of his injury resulting in gastric perforation [13].

## Conclusions

Esophageal perforation can occur as a rare but serious complication of blind nasogastric tube advancement, even when initial radiographic positioning appears appropriate. This case highlights the risk of submucosal esophageal tracking with distal gastric perforation in patients with altered esophageal integrity, particularly following chemoradiation and dilation. Early recognition of clinical deterioration and prompt imaging are critical for diagnosis. In high-risk patients, consideration of image-guided or direct visualization techniques during tube placement may help reduce the risk of occult malposition and severe iatrogenic injury.

## References

[1] Subedi Y, Adhikari B, Pokharel A, Poudel K, Sharma S (2023). A case report of esophageal perforation: complications of orogastric tube placement. Cureus.

[2] Numata Y, Ishii K, Seki H, Yasui N, Sakata M, Shimada A, Matsumoto H (2018). Perforation of abdominal esophagus following nasogastric feeding tube intubation: a case report. Int J Surg Case Rep.

[3] Isik A, Firat D, Peker K, Sayar I, Idiz O, Soytürk M (2014). A case report of esophageal perforation: complication of nasogastric tube placement. Am J Case Rep.

[4] Kaman L, Iqbal J, Kundil B, Kochhar R (2010). Management of esophageal perforation in adults. Gastroenterology Res.

[5] Guthrie DB, Pezzollo JP, Lam DK, Epstein RH (2020). Tracheopulmonary complications of a malpositioned nasogastric tube. Anesth Prog.

[6] Gidda H, Mansour M, Singh I, Nashed B, Ventimiglia W (2023). The forgotten complication of nasogastric tube insertion: esophageal perforation and associated hydropneumothorax and hydropneumoperitoneum. Cureus.

[7] Merzouqi B, El Bouhmadi K, Zouhair N (2021). A rare cause of cervicomediastinal cellulitis: oesophageal perforation case report. Ann Med Surg (Lond).

[8] Abila AW, Nditika ME, Kipkemoi RD, Ondigo S, Khwa-Otsyula BO (2020). Primary repair of esophageal perforation: case report. Int J Surg Case Rep.

[9] Shaheem S, Panikkaveettil H (2024). Aetiology, clinical manifestations, diagnosis, and treatment of oesophageal perforation: a review. Cureus.

[10] Lampridis S, Mitsos S, Hayward M, Lawrence D, Panagiotopoulos N (2020). The insidious presentation and challenging management of esophageal perforation following diagnostic and therapeutic interventions. J Thorac Dis.

[11] Søreide JA, Viste A (2011). Esophageal perforation: diagnostic work-up and clinical decision-making in the first 24 hours. Scand J Trauma Resusc Emerg Med.

[12] Mzoughi Z, Djebbi A, Bayar R, Hamdi I, Gharbi L, Khalfallah T (2016). Traumatic isolated perforation of lower oesophagus. Trauma Case Rep.

[13] Eroglu A, Aydin Y, Ulas AB (2022). Minimally invasive and endoscopic approach to esophageal perforation. Eurasian J Med.

